# Predictors of Regional Lymph Node Recurrence after Initial Thyroidectomy in Patients with Thyroid Cancer

**DOI:** 10.1155/2016/4127278

**Published:** 2016-06-14

**Authors:** Amirsina Sharifi, Abolfazl Shojaeifard, Ahmadreza Soroush, Mehdi Jafari, Ali Ghorbani Abdehgah, Hossein Mahmoudzade

**Affiliations:** Department of General Surgery, Shariati Hospital, North Kargar Street, Tehran, Iran

## Abstract

*Background.* Regional lymph node recurrence (RLNR) is common in patients with thyroid cancer but clinicopathological predictors are unclear. We aimed to clarify these predictors and identify patients who would benefit from prophylactic lymph node dissection the most.* Method.* 343 patients with different types of thyroid cancer were analyzed retrospectively. All patients underwent total thyroidectomy between 2007 and 2013.* Results.* The median ± interquartile range of patients' age was 40 ± 25 years. 245 (71.4%) patients were female. Regarding the risk of regional lymph node recurrence, we found that male gender, age ≥45 years, non-PTC (i.e., medullary, follicular, and anaplastic types) histopathology, T3 (i.e., tumor size >4 cm in the greatest dimension limited to the thyroid or any tumor with minimal extrathyroid extension), stage IVa, and isolated cervical lymphadenopathy as initial manifestation (ICL) are significant risk factors. T3 (*p* < 0.001; odds ratio = 156.41, 95% CI [55.72–439.1]) and ICL (*p* < 0.001; odds ratio = 77.79, 95% CI [31.55–191.81]) were the strongest predictors of regional lymph node recurrence.* Conclusion.* We found easily achievable risk factors for RLNR in thyroid cancers patients. We suggested that patients with specific clinicopathological features like male gender, age ≥45 years, larger tumor size, and extrathyroidal extension be considered as prophylactic lymphadenectomy candidates.

## 1. Introduction

Thyroid cancer is the most common cancer of the endocrine system with growing incidence all over the world. Thyroid cancers cause less than 0.5% of cancer-caused deaths. Most types of thyroid cancer are known to be differentiated with slow clinical progression and cause occasional distant metastasis in patients [1–3]. On the other hand, regional metastasis to cervical lymph nodes is found in about 70% of thyroid cancers as a part of their clinical development. Regional metastasis to cervical lymph nodes is an independent risk factor of recurrence; although the effect of recurrence on decrement of survival is clearly acceptable, this subject remained a controversy [4, 5]. It is believed that recurrence plays an important role in clinical manifestation of differentiated thyroid cancers. Previous studies demonstrated different risk factors that influence recurrence of differentiated thyroid cancer: age, tumor size, histological features of the tumor, and regional and distant metastasis [6–8]. Based on a recent study by Wada et al., patients with regional metastasis to cervical lymph nodes have a recurrence rate of 16.3% while in those without metastasis recurrence rate is zero percent [9]. There are different widespread staging systems to predict prognosis of differentiated thyroid cancers. Nevertheless, some of these systems did not even consider the effect of lymph node involvement and once this effect is included it is based on the anatomical region of lymph nodes and the presence of involvement, not on the basis of histological diagnosis of engagement severity [10–12]. For example, in AJCC scoring system, patients with lymph node metastasis are categorized in N1a and N1b subgroup. In this system, a patient with 2/48 of total cervical lymph nodes is grouped with a patient having 46/48 of metastatic lymph nodes [10]. Considering the fact that the importance and necessity of surgical treatment of lymph nodes is not clearly defined, it is necessary to evaluate the effect of regional lymph node recurrence on patients' outcome. In this study, we aimed to investigate this effect and the clinicopathological features indicating prophylactic lymphadenectomy in order to reduce recurrence rate.

## 2. Method

We retrospectively reviewed medical records of 343 patients referred to the general surgery ward of Shariati Hospital, Tehran University of Medical Sciences (TUMS), Tehran, Iran, between March 2007 and March 2013, who underwent thyroidectomy to any extent due to thyroid cancer. Patients managed in other institutions, as well as their pathological specimens, who were referred to our center for second expert opinion were excluded. Required information was collected using the database of the pathology ward of Shariati Hospital and the following data of patients who met the inclusion criteria were gathered: age, gender, anatomical site of tumor, tumor size and weight, presence of single or multinodular goiter, histological type of thyroid cancer, presence of either capsular or vascular or perineural involvement, and regional lymph node metastasis. The ethnic committee of Shariati Hospital approved our study protocol.

All patients with node-positive PTC or other node-positive thyroid cancers had total thyroidectomy with systematic lymph node dissection of the central neck compartment. The other lymph nodes were dissected as if they were clinically involved. All operations were conducted by a single surgical team in our center.

Thyroid-stimulating hormone suppression therapy was set for all the patients after surgery, and those who were diagnosed with LN metastasis, capsular invasion, or extrathyroidal extension according to pathological reports of thyroid specimen received additional postsurgical radioactive iodine treatment. ^131^I ablative therapy was administered after levothyroxine withdrawal for at least 4 weeks. A whole body scan (WBS) was performed 7 days after ^131^I therapy in order to evaluate whether ablation was successful. Patients received a routine periodic clinical examination every 3 months in the first year and then at 6-month intervals for the following year and then at yearly intervals. Each visit consists of neck ultrasound, WBS, measurement of serum free T4, thyroid-stimulating hormone (TSH), thyroglobulin (Tg), and anti-Tg antibodies.

All patients were categorized based on TNM classification.

The node recurrence was defined as either pathologically approved disease on excision or cytology or recurrent disease confirmed by elevated Tg and whole body scan (WBS) in patients that underwent total thyroidectomy.

All clinicopathological factors potentially known to have an effect on regional recurrence were assessed using univariate analysis using Fisher's exact or chi-squared tests. Significant variables in the univariate analysis were examined in multivariate analysis using a binary logistic regression test. SPSS version 16.0 software (SPSS Inc., Chicago, IL) was used for statistical analysis. Statistical significance was defined as a *p* value < 0.05.

## 3. Results

The clinicopathological history of 343 patients with thyroid cancer was reviewed. The median ± interquartile range (IQR) of participants' age was 40 ± 25 years, and 245 (71.4%) patients were female. Demographic and clinicopathological findings are represented in Tables 1, 2, and 3. Cancer histopathology of the majority of patients (86.3%) was papillary thyroid carcinoma (PTC), and also most of the patients (57.1%) were in stage I. Also, 76.9% of the patients had multinodular goiter (MNG). Capsular invasion, vascular invasion, and perineural invasion were found in 60.4%, 16.1%, and 21.9% of the patients, respectively. Moreover, regional lymph node recurrence occurred in approximately half of the patients (49.6%). Only 12 (3.5%) of patients showed distant metastasis at their initial presentation.

Cancer pathology was significantly associated with disease stage; so medullary, follicular, and anaplastic types were more associated with advanced stages (*p* < 0.0001, Cramer's *V* = 0.383). Also, patients with follicular thyroid cancer (FTC) were approximately 15 years older than PTC patients (*p* = 0.018). Stage I patients were significantly younger than patients with higher stages (*p* < 0.0001). Male gender was found to be more common among medullary, follicular, and anaplastic types (*p* = 0.003; odds ratio = 2.511, 95% CI [1.337–4.717]). Regarding microscopic tumor invasion, vascular invasion was found more commonly in medullary, follicular, and anaplastic types (*p* = 0.013; odds ratio = 4.792, 95% CI [1.488–15.428]). Also, binary logistic regression showed us that the risk of capsular invasion increased by 2.7% (95% CI: 1–5.5%) along with each one-year increase in the age (*p* = 0.049). Also, the risk of perineural invasion increased by 7.9% (95% CI: 1.1–15.2%) along with each one-year increase in the age (*p* = 0.022). MNG was significantly correlated with female gender (*p* = 0.034; odds ratio = 2.384, 95% CI [1.052–5.402]). Metastasis (M1) was more likely to happen in medullary, follicular, and anaplastic types (*p* = 0.003; odds ratio = 6.902, 95% CI [2.125–22.419]). Also, we observed that each one-year increase in age was associated with 5.5% (95% CI: 1.8–9.3%) increase in the risk of metastasis (*p* = 0.004).

Regarding the risk of regional lymph node recurrence, we observed that male gender, age ≥45 years, non-PTC (i.e., medullary, follicular, and anaplastic types) histopathology, T3 (i.e., tumor more than 4 cm in the greatest dimension limited to the thyroid or any tumor with minimal extrathyroid extension such as extension to sternothyroid muscle or perithyroid soft tissues), stage IVa, and isolated cervical LAP as initial disease manifestation are significant risk factors. Among these risk factors, T3 (*p* < 0.001; odds ratio = 156.41, 95% CI [55.72–439.1]) and isolated cervical LAP at initial presentation (*p* < 0.001; odds ratio = 77.79, 95% CI [31.55–191.81]) were the strongest predictors of regional lymph node recurrence.

## 4. Discussion

Although the overall prognosis of PTC is good, local recurrence after initial surgery stays an issue; also, in other thyroid cancers, clinicopathological factors predicting local recurrences are unclear [13]. In this regard, performing prophylactic lymph node dissection is a controversy [14, 15]. Some authors advised lymph node dissection because of a reduction in postoperative thyroglobulin (Tg) levels and also a decrease in morbidity rate which is obtained in the first operation [16–18] and other investigators suggested that this procedure increases the risk of injury to local tissues like parathyroid glands and recurrent laryngeal nerves without any significant effect on survival [19–22]. Thus, clinicopathological predictors of greater recurrence risk in lymph node would be valuable.

In this study, we found that male gender, age ≥45 years, non-PTC histopathology, and T3 stage are significant risk factors of regional lymph node recurrence. Among these risk factors, T3 stage (*p* < 0.001; odds ratio = 156.41, 95% CI [55.72–439.1]) and isolated cervical LAP at initial presentation (*p* < 0.001; odds ratio = 77.79, 95% CI [31.55–191.81]) were the strongest predictors of regional lymph node recurrence.

Previous studies proposed that male gender, large tumor size, extrathyroidal extension, capsular invasion, and lymphovascular invasion are predictors of both prognosis in thyroid cancers [23] and risk factors for central lymph node metastasis [24]. These findings are comparable to ours.

Qu et al. showed that, in a group of 514 male and 2128 female patients, 26.3% of the patients with regional lymph node recurrence were male against 15% of the patients without [24].

Studies investigating the effect of age in RLNR in patients with PTC suggested that age <20 years and age >70 years have a poor prognosis independent of cancer stage [25, 26]. In our study, the incidence of regional lymph node recurrence was higher in patients aged ≥45 years.

It is believed that large tumor size at presentation is associated with higher risk of regional recurrence [27, 28]. Tumor size greater than 0.5 cm in diameter has been proposed to strongly be associated with regional lymph node recurrence [29, 30]. We reported that tumor size >4 cm in the greatest dimension is more likely to precede regional lymph node recurrence.

The effect of extrathyroidal extension has been investigated in some studies and the elevated risk of regional lymph node recurrence has been reported as a result of capsular invasion, vascular invasion, and perineural invasion [31, 32]. Scheumann and colleagues reported that extrathyroid extension or higher T stage is associated with higher rate of local recurrence and lower survival rate, as well as an increase in lymph node recurrence [33]. We showed that patients with T3 stage based on TNM classification are at greater risks for developing regional lymph node recurrence.

Baek et al., based on multivariate analysis results, stated that only the N stage was significantly related to regional recurrence, and the disease-free survival period was significantly shorter in the lymph node metastasis-positive group than in the lymph node metastasis-negative group [34]. We found cervical LAP at initial presentation, one of the strongest predictors of regional lymph node recurrence, to be a significant risk factor.

The strongest predictor of regional lymph node recurrence was tumor size more than 4 cm and extrathyroid extension. Tumor cells generally spread through the lymphatic system from the thyroid gland to the central and lateral compartments [35]. Skip metastasis to the lateral compartment of the neck in the absence of central disease is uncommon [35].

This study has limitations. Our study was performed retrospectively and not all patients had the same follow-up period. Also, our criteria for diagnosis of regional lymph node recurrence were not completely capable of finding each recurrence so we might have missed some cases.

## 5. Conclusion

Our study revealed several risk factors for secondary LNM in patients with thyroid cancers, all of which are easily achievable in clinical settings. The identification of patients with greater risks of secondary LNM has a great impact on their survival. In this regard, a group of patients who undergo prophylactic lymphadenectomy ultimately have better outcomes. We suggested that patients with specific clinicopathologic features like male gender, larger tumor size, and extrathyroidal extension be considered as candidates for prophylactic lymphadenectomy.

## Figures and Tables

**Table 1:** Demographic and clinicopathological characteristics.

Characteristics	Count (%)
Gender	
Female	245 (71.4%)
Male	98 (28.6%)

Disease manifestation	
Isolated thyroid nodule	141 (44.31%)
Thyroid nodule + cervical LAP	64 (20.3%)
Isolated cervical LAP	102 (32.3%)
Central cervical LAP	1 (0.98%)
Unilateral cervical LAP	39 (38.235%)
Bilateral cervical LAP	52 (50.98%)
Central + (unilateral or bilateral) cervical LAP	4 (3.922%)
Submandibular LAP	6 (5.882%)
Distant lymph node metastasis	9 (2.8%)
Supraclavicular LAP	3 (0.9%)
Subclavicular LAP	2 (0.6%)
Mediastinal LAP	4 (1.3%)
Skin metastasis	1 (0.316%)

Cervical lymphadenopathy	
NA	120 (35.0%)
No cervical LAP	31 (9.0%)
Paratracheal LAP	5 (1.5%)
Pretracheal or central LAP	25 (7.3%)
Unilateral, bilateral, or contralateral cervical LAP	152 (44.3%)
Retropharyngeal or superior mediastinal LAP	4 (1.2%)
Distant lymph node metastasis	6 (1.7%)

**Table 2:** Demographic and clinicopathological characteristics.

Characteristics	Count (%)
Goiter	
Single	36 (23.1%)
Multinodular	120 (76.9%)

T	
T1	61 (17.8%)
T2	22 (6.4%)
T3	195 (56.9%)
T4a	43 (12.5%)
T4b	22 (6.4%)

N	
Nx	126 (36.7%)
N0	31 (9.0%)
N1a	30 (8.7%)
N1b	156 (45.5%)

M	
Mx or M0	331 (96.5%)
M1	12 (3.5%)

Capsular invasion	
No	38 (39.6%)
Yes	58 (60.4%)

Vascular invasion	
No	125 (83.9%)
Yes	24 (16.1%)

Perineural invasion	
No	25 (78.1%)
Yes	7 (21.9%)

Lymph node metastasis (LNM)	
No LNM	65 (27.5%)
LNM at initial presentation	54 (22.9%)
Regional lymph node recurrence	117 (49.6%)

**Table 3:** Demographic and clinicopathological characteristics.

Characteristics	Count (%)
Cancer type	
Papillary thyroid carcinoma (PTC)	289 (86.3%)
Medullary thyroid carcinoma (MTC)	26 (7.8%)
Follicular thyroid carcinoma (FTC) or Hürthle cell carcinoma (HCC)	16 (4.8%)
Anaplastic (undifferentiated) thyroid carcinoma (ATC or UTC)	4 (1.2%)
Stage	
I	192 (57.1%)
II	15 (4.5%)
III	29 (8.6%)
IVa	81 (24.1%)
IVb	11 (3.3%)
IVc	8 (2.4%)
Capsule status	
Uncapsulated	70 (55.1%)
Capsulated	31 (24.4%)
Partially capsulated	26 (20.5%)
